# A review of the current treatment methods for retroperitoneal fibrosis with obstructive uropathy

**DOI:** 10.1002/bco2.371

**Published:** 2024-05-12

**Authors:** Charles Carey, Gerard Gurumurthy, Richard Napier‐Hemy, Bachar Zelhof

**Affiliations:** ^1^ Manchester University NHS Foundation Trust Manchester UK; ^2^ The University of Manchester Manchester UK

**Keywords:** hydronephrosis, literature review, management, obstructive uropathy, retroperitoneal fibrosis

## Abstract

**Introduction and aims:**

Retroperitoneal fibrosis (RPF) is a fibroinflammatory disease in which patients may suffer obstructive uropathy (OU). The optimum treatment strategy for RPF with secondary OU is currently unclear, and the aim of this literature review is to assess the methods used to treat this patient cohort.

**Methods:**

Medline, Embase, Cinahl, the Cochrane Library and PubMed were systematically searched to find studies assessing treatment outcomes in this patient cohort. After reviewing the studies' titles, abstracts and full texts, 12 were found that matched our search aims. Data from these publications were analysed and reported.

**Results:**

The demographic and symptomatic features of patients across the 12 studies were representative of the general RPF population. No randomised control trials (RCTs) were found, and just one study formally compared outcomes between patients who underwent different treatment strategies. Many of the studies concluded that using medical and surgical methods in combination led to positive outcomes; whereas, others found positive outcomes following a variety of regimens. Many studies also highlighted, however, that significant minorities required further treatment after initial therapy. Conclusions regarding optimum treatment methods were limited as most publications did not formally compare outcomes following different strategies and had an observational study design.

**Conclusion:**

Although positive outcomes were commonly seen following medical, surgical and a combination of treatments, the literature currently lacks research formally comparing outcomes after assigning specific treatment protocols to groups of RPF patients. More research is therefore required to determine how to best manage RPF leading to secondary OU.

## 1 INTRODUCTION AND AIMS

Retroperitoneal fibrosis (RPF) is a rare fibroinflammatory disease that results in the inflammation and fibrosis of adventitial tissue. This occurs around the aorta and common iliac vessels, which spreads to the peri‐adventitial space.^1^, ^2^ As a consequence, many patients experience compression and subsequent dysfunction of the retroperitoneal organs, manifesting as obstructive uropathy (OU) and necessitating urological interventions.^2^

Several studies have investigated the outcomes of different treatment modalities, mainly involving corticosteroids, immunosuppressive agents and tamoxifen.^1^ Surgical interventions such as ureteric stenting, nephrostomy and ureterolysis are also used to manage RPF and its complications. Currently, there is no consensus on the optimal management of these patients, and the choice of treatment is often decided based on disease severity and the presence of complications.

Although the role of IgG4 in the aetiology of RPF is becoming better understood, only a fraction of patients present with this antibody, and it cannot explain why the condition arises in all cases.^3^, ^4^ Furthermore, the exact role of IgG4 in the fibroinflammatory process that occurs in RPF remains unclear. It may be true that those displaying positive IgG4 serology respond differently to RPF treatments than IgG4 negative RPF patients.

Given the complexity of the disease and lack of homogeneity in the choice of treatments, a review of the current literature was necessary to determine the optimal treatment options for RPF patients with secondary OU. This review describes the common ways in which this condition presents and investigates the current methods used to optimise kidney drainage and minimise the use of synthetic materials.

## 2 METHODS

**FIGURE A1:**
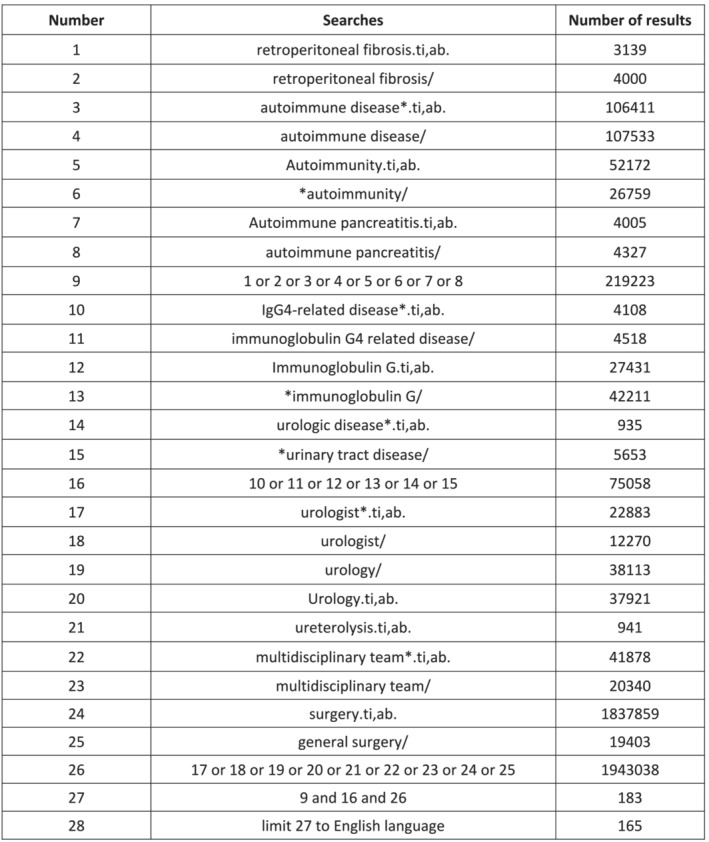
Literature search strategy.

A literature search for studies published between 2009 and 2022 that compared methods of facilitating kidney drainage in patients with urinary obstruction secondary to RPF was performed. Medline, Embase, Cinahl and the Cochrane Library were searched using the strategy outlined in Figure A1. A separate search using the PubMed Advanced Search Builder with the terms ‘retroperitoneal fibrosis’ AND ‘IgG4’ AND ‘surgery’ was also performed. Following the removal of duplicate papers, 611 studies were included for an abstract review in our initial search. Studies that were irrelevant to the subject matter, where patients had no clear evidence of urinary obstruction, review articles, case reports and abstracts linked to poster presentations, were excluded. Studies with abstracts not written in English were also excluded.

Following a review of titles and abstracts, 385 studies were excluded as they were deemed irrelevant, and the remaining 226 were included for a full text review. Subsequent assessment of the studies led to the exclusion of 174 publications and analysis of 52 studies, which included 33 primary studies, 19 case reports and case series. Case reports and irrelevant primary studies were then removed, and 12 remaining studies were ultimately included and analysed.

## 3 RESULTS

### 3.1 Patient demographics and clinical presentation

The patient demographics from the studies included are shown in Table 1. The studies came from a variety of countries across Europe, North America, South America and Asia, and 430 patients were included in total. The median (interquartile range [IQR]) number of patients included in the studies was 25 (23.5). The median (IQR) age and proportion of males among all the patients studied were 56.7 (5.9) years and 70% (14.3), respectively. The studies therefore included patients representative of the typical RPF cohort, which mostly consists of male patients aged between 40 and 60 years.^1^ Eleven out of the 12 studies included information regarding 17 presenting signs and symptoms, which are summarised in Table 2. Renal failure, loin pain and abdominal pain were the most common symptoms reported, with frequencies of 61.6%, 54.7% and 38.2%, respectively. The treatment methods employed across the studies are summarised in Table 3. Three studies mentioned using either an open or laparoscopic approach for ureterolysis, and none reported performing this procedure with robotic assistance.

**TABLE 1:** Demographics of the patients in the studies included.

Authors	Number of patients included	Average age of patients in study (years)	Percentage of males in study	Study design	Average length of follow‐up in months (range)	Hydronephrosis, hydroureter and/or ureteric obstruction (%)
Liu et al.^5^	58	60	63.8	Retrospective, single centre observational	24 (1–109)	60.3
Santiago et al.^6^	52	56	65	Retrospective, single centre observational	50.4	100
Zhou et al.^7^	30	56.7	76.7	Retrospective, single centre observational	30 (4–136)	86.7
Liu et al.^8^	17	56	75	Retrospective, single centre observational	5 (3–13)	100
Bilgo et al.^9^	18	51.4	61.1	Retrospective, single centre observational	72	87.5
Bergero et al.^10^	5	60.4	20	Retrospective single centre observational	31.2 (25–63)	100
Liao et al.^11^	142	54.3	84.5	Retrospective, single centre observational	Length of follow‐up not stated	72.5
Yachoui et al.^12^	26	58	76.9	Retrospective, single centre observational	60 (25.2–84)	65.4
Liu et al.^13^	30	65.9	76.7	Retrospective, single centre observational	Length of follow‐up not stated	100
Chua et al.^14^	24	54.5	62.5	Retrospective, single centre observational	Length of follow‐up not stated	‐
Forestier et al.^15^	18	Median values quoted for 3 separate groups	77	Retrospective, single centre observational	Follow‐up times values quoted for 3 separate groups	61.1
Chiba et al.^19^	10	70.1	62.5	Retrospective, single centre observational	14.5 (6–60)	50.0

*Note*: Table 1 shows the mean ages, proportions of male patients included and percentages presenting with hydronephrosis and/or hydroureter across the 12 studies. On average, the patients were aged between 50 and 70, and the median percentage of males was 70 (IQR = 14.35). Of note is that seven studies quoted a mean age and five quoted a median age. One study quoted a median age for 3 separate patient groups. All the studies included patients with documented hydronephrosis except for Chua et al., in which all patients underwent ureteric stenting. The studies are listed in the order in which they are discussed below.

**TABLE 2:** Presenting features of the patients in the studies included.

Authors	Number of patients	Asymptomatic (%)	Abdominal pain (%)	Loin pain (%)	Back pain (%)	Renal failure (%)	Fatigue (%)	Lower limb oedema (%)	Weight loss (%)	Nausea and vomiting (%)	Arthralgia (%)	Oliguria (%)	Scrotal issues (%)	Fever	Diarrhoea	Constipation	Weight loss	Hypertension	Haematuria
Liu et al.^5^	58	‐	24.1	48.3	‐	75.9	8.6	61.8	‐	6.9	‐	‐	‐	5.2	‐	‐	‐	24.1	‐
Santiago et al.^6^	52	‐	‐	‐	‐	‐	‐	‐	‐	‐	‐	‐	‐	‐	‐	‐	‐	‐	‐
Zhou et al.^7^	30	13.3	13.3	26.7	13.3	‐	20.0	13.3	30.0	16.7	‐	13.3	3.3	‐	‐	‐	30.0	53.3	6.7
Liu et al.^8^	17	52.9	35.3	‐	‐	‐	‐	17.6	‐	‐	‐	11.8	‐	‐	5.9	5.9	‐	‐	‐
Bilgo et al.^9^	18	‐	‐	77.7	‐	83.3	‐	11.1	16.7	‐	‐	11.1	22.2	16.7	‐	‐	‐	‐	‐
Bergero et al.^10^	5	‐	54.2	‐	60	‐	20	‐	‐	‐	‐	40	‐	‐	‐	‐	20	‐	‐
Liao et al.^11^	142	‐	54.2	66.9	13.4	50.7	‐	22.5	26.8	12.7	‐	‐	3.5	11.3	3.5	16.2	26.3	‐	2.8
Yachoui et al.^12^	26	8.3	58.3	41.7	29.2	45.8	‐	4.2	‐	16.7	4.2	‐	‐	‐	4.2	12.5	‐	‐	‐
C‐H. Liu et al.^13^	30	‐	‐	‐	‐	‐	‐	‐	‐	‐	‐	‐	‐	‐	‐	‐	‐	‐	‐
Chua et al.^14^	24	‐	‐	‐	‐	91.7	‐	‐	‐	‐	‐	‐	‐	‐	‐	‐	‐	8.3	‐
Forestier et al.^15^	18	‐	27.8	66.6	‐	22.2	‐	27.8	‐	‐	‐	‐	27.8	‐	‐	5.6	‐	‐	‐
Chiba et al.^19^	10	‐	‐	‐	10	‐	‐	10	‐	‐	10	‐	‐	10	‐	‐	‐	‐	‐

*Note*: Table 2 displays the percentages of patients included who reported 17 signs and symptoms. All studies published data related to presenting symptoms except for Santiago et al. Renal failure, abdominal and loin pain were the most common symptoms reported. Studies reporting lumbar or flank pain were included in the loin pain column. Values were included in the renal failure column if the study quoted a number/percentage of patients who displayed renal failure, impaired renal function and/or acute kidney injury. Symptoms that were not reported are denoted by a dash. Although the study by Yachoui et al. included 26 patients, they reported symptoms for 24 patients as two had missing data.

**TABLE 3:** Treatment methods employed across the studies.

Authors	Number of patients included	Number (%) treated with medications	Number (%) treated surgically
Liu et al.^5^	58	Corticosteroids—29 (50%) Cyclophosphamide—19 (65.5%) Tamoxifen—2 (6.9%) Leunomide—1 (3.5%)	Double‐J ureteric stent insertion and ureterolysis—37 (88.1%) Endovascular stent implantation into the iliac artery—2 (4.8%) Ureteric lesion excision—1 (2.4%) Resection of a neoplasma surrounding the abdominal aorta—1 (2.4%) Inguinal lymph node biopsy—1 (2.4%)
Santiago et al.^6^	52	Corticosteroids alone—3 (5.8%) Corticosteroids and steroid sparing agent—47 (90.4%) Steroid sparing agents alone—2 (3.8%)	Ureteric stent insertion—50 (96.2%) Ureterolysis due to medication intolerance—8 (15.4%) Nephrectomy—4 (7.6%) Percutaneous nephrostomy following stent failure—3 (5.8%)
Zhou et al.^7^	30	Corticosteroids alone—15 (50.0%) Corticosteroids and cyclophosphamide—1 (3.3%) Corticosteroids and tamoxifen—8 (26.7%) Tamoxifen alone—1 (3.3%) Haemodialysis—3 (10.0%)	Double‐J ureteric stent insertion—26 (86.7%) Metallic stent insertion—1 (3.3) Percutaneous nephrostomy—2 (6.7%) Open ureterolysis and ureter intraperitonealisation—5 (16.7%) Laparoscopic ureterolysis and ureter intraperitonealisation—5 (16.7%)
Liu et al.^8^	17	Corticosteroids – 17 (100%)	Ureteric stent insertion—13 (76.5%) Ureterolysis 3 (17.4%)—performed after failed ureteric stent insertion
Bilgo et al.^9^	18	Corticosteroids alone—12 (66.7%) Corticosteroids and Immunosuppressants—1 (5.6%) Corticosteroids and tamoxifen—1 (5.6%)	Double‐J ureteric stent insertion—18 (100%) Open ureterolysis and ureter intraperitonealisation—3 (16.7%) Nephrostomy—1 (5.6%)
Bergero et al.^10^	5	Corticosteroids—4 (80%) Tamoxifen—2 (40%)	Laparoscopic ureterolysis with ureter intraperitonealisation without omentoplasty—5 (100%)
Liao et al.^11^	142	Corticosteroids alone—7 (4.9%) Corticosteroids and cyclophosphamide—48 (33.8%) Corticosteroids and leflunomide—16 (11.3%) Corticosteroids and methotrexate—2 (1.4%) Corticosteroids and azathioprine—3 (2.1%) Corticosteroids and mycophenolate mofetil—4 (2.8%) Corticosteroids and tamoxifen—2 (1.4%) Corticosteroids and two or more immunosuppressants—27 (19.0%) Corticosteroids and rituximab—1 (0.7%) Hydroxychloroquine alone—2 (1.4%)	Ureteric stent insertion—69 (48.6%). 5 received this intervention before needing an acute admission. Ureterolysis—9 (6.3%) Percutaneous nephrostomy—6 (4.2%) Nephrectomy—3 (2.1%)
Yachoui et al.^12^	26	Corticosteroids alone—10 (38.5%) Corticosteroids and azathioprine—1 (3.8%) Corticosteroids and mycophenolate mofetil – 1 (3.8%) Corticosteroids and methotrexate—1 (3.8%) Tamoxifen alone—3 (11.5%) Corticosteroids and tamoxifen—2 (7.7%) No treatment—1 (3.8%)	Double‐J ureteric stent insertion—14 (53.8%). Bilateral—7 (26.9%) Unilateral—7 (26.3%) Percutaneous nephrostomy—2 (87.7%). Unilateral—1 (3.8%) Bilateral—1 (3.8%) Unilateral—1 (3.8%) Ureterolysis—7 (26.9%) Unilateral—6 (23.1%) Bilateral—1 (3.8%) Vascular stenting—1 (3.8%) Other surgery—3 (11.5%)
Liu et al.^13^	30	Corticosteroids only—3 (10.0%) Corticosteroids and immunomodulators—2 (6.6%)	Double‐J ureteric stent insertion—11 (36.7%) Metallic ureteric stent insertion—6 (20%) Ileal reconstruction—4 (13.3%) Ureterolysis—2 (6.7%) Percutaneous nephrostomy—1 (3.3%)
Chua et al.^14^	24	Azathioprine—7 (29.2%) Corticosteroids, methotrexate and rituximab were used in an unspecified number of patients	Double‐J ureteric stent insertion or nephrostomy—24 (100%) Extra anatomical stenting—2 (8.3%) Ureterolysis—17 (70.8%) Nephrectomy—5 (20.8%)
Forestier et al.^15^	18	Only the patients with possible and highly suggestive IgG4 related RPF had their treatment described. Corticosteroids alone—12 (100%) Corticosteroids and azathioprine—2 (16.7%) Corticosteroids and methotrexate—2 (16.7%)	Bilateral ureteric stent insertion—2 (16.7%)
Chiba et al.^19^	10	Corticosteroids—7 (70.0%)	Double J ureteric stent insertion—1 (10.0%) Resection of a ureteric mass—1 (10.0%) Resection of a periaortic aneurysm—10 (10.0%)

*Note*: Table 3 shows the different treatments received by the patients in each study. Overall corticosteroids and ureteric stent insertion were employed most frequently. Although the combinations of medical treatments received were usually described, generally, the studies did not reveal whether treatments were given simultaneously or sequentially. While the studies usually commented on how many received various forms of surgery, it was commonly challenging to determine how surgery was combined with medical treatments. Patients who underwent more than one form of surgery during their treatment have these counted separately.

### 3.2 Treatments and outcomes

The study by Liu et al. was the only investigation that directly compared the outcomes of patients who received medical, surgical or a combination of medical and surgical treatments. Forty‐two patients were treated surgically, of which 37 (88.1%) underwent double‐J stent insertion and ureterolysis and 2 (4.8%) received endovascular stenting.^5^ Excision of a ureteric lesion, neoplasm excision and inguinal node biopsy were also performed once in three separate patients.^5^ Corticosteroids were given to all 29 patients who received medical treatments alone and 22 later received treatment with an immunosuppressant.^5^ A subsection of these two treatment groups consisting of 17 patients received both surgery and corticosteroids.

Overall, the study showed high rates of treatment success, with 79.3% showing symptomatic improvement.^5^ In addition, double‐J stents were removed indefinitely after an average of 2.5 stent changes, and just five (17.2%) patients relapsed after stopping corticosteroids.^5^ Significant improvements were seen after 12 months in all three treatment cohorts.^5^ Combination therapy, however, was associated with a higher percentage of patients with a good prognosis after at least 2 years of follow‐up compared with those who received surgery.^5^ Because the exact figures representing outcomes for the surgical patients who did not receive medical treatment were not reported, the exact benefits of combination treatment versus surgery alone could not be evaluated. Furthermore, because patients received several different medications, this study does not analyse the benefits of individual regimens.^5^ These reasons in addition to the study's retrospective designs and small sample sizes limit the extent of their conclusion that combination therapy is superior to surgery alone.

### 3.3 Studies where medical and surgical treatments were indirectly compared

Santiago et al. performed a study in which all patients received medical treatment and 94.4% underwent ureteric stenting.^6^ Thirty‐six (69.2%) patients were able to avoid further surgery after stent insertion, and nine (17.3%) suffered disease relapse. Eight of those who suffered relapse underwent subsequent ureterolysis.^6^ This additional procedure failed to prevent further relapse in three cases. After 7‐year follow‐up, eight (15.4%) patients required either a nephrectomy or long‐term stent insertion.^6^ Because a large proportion of the patients were able to avoid major surgery following the concurrent use medications and ureteric stenting, this study suggests that combining these treatment methods is often highly effective. A significant minority relapsed following while using this combination, however, and so frequent long‐term follow‐up is likely to be needed so that further interventions may be initiated promptly. The study also showed that there were no statistically significant differences between the inflammatory and renal function markers between patients treated successfully with medications and stenting, who required subsequent ureterolysis or who experienced a morbid event before or after treatment.^6^ These parameters were therefore unable to predict future treatment success.

In a study of 30 patients by Zhou et al., 26 (86.7%) received double‐J stent insertion and immunomodulators. Five of these patients were made permanently stent free, one required metallic stent insertion, and it is implied that the other 20 underwent regular stent replacement during the follow‐up period.^7^ Fifteen (50%) patients received corticosteroids, eight (26.7%) received corticosteroids with tamoxifen and one (3.3%) received corticosteroids with immunomodulators. The majority therefore received a combination of ureteric stenting and medications.^7^ Alternative procedures were performed in 50% of patients, with two undergoing percutaneous nephrostomy insertion, three receiving haemodialysis and 10 requiring ureterolysis.^7^ Because the treatment timings and exact combinations are unclear, it is not possible to determine whether these additional treatments were required before, after or instead of ureteric stent insertion. Because the number of operations also exceeded the number of patients in the study, some patients will have received more than one procedure.^7^ It is possible that this was required due to treatment failure or relapse, though this is not stated explicitly in the study.^7^

Zhou et al. showed that the treatment strategies employed improved the cohort's average erythrocyte sedimentation rate (ESR), C‐reactive protein (CRP) and retroperitoneal mass sizes.^7^ The treatments were therefore effective to an extent, but there were also 21 cases where ureteric stents could not be permanently removed.^7^ Because many are likely to have received stenting and/or ureterolysis with medical treatment, this study suggests that combination therapy can be effective in some and limited in others. The strength of this conclusion is limited however because the number of patients receiving different treatment combinations was not reported and outcomes between treatment groups were not compared.

Another study reported outcomes in a cohort of 17 RPF patients with hydronephrosis who all received steroid treatment.^8^ Four (23.5%) patients did not require any procedures to facilitate kidney drainage, and the remaining 13 (76.5%) underwent ureteric stenting before starting medications.^8^ Three (23.1%) who underwent stenting required subsequent ureterolysis, and nine (69.2%) were able to have their stents removed without further surgery when assessed at follow‐up.^8^ This study suggests that combining ureteric stent insertion with steroid treatment can result in many being stent‐free during follow‐up, mirroring the findings by Liu et al. and Santiago et al. The study also suggests, however, that others will require ureterolysis after ureteric stent insertion and so that all patients should receive close and regular follow‐up.

Bilgo et al. also reported good overall outcomes among a cohort of 18 RPF patients with OU.^9^ All 18 patients in this study underwent double‐J stent insertion, and 12 (66.7%) additionally received corticosteroids either alone or with tamoxifen and immunosuppressants.^9^ The exact timings of these interventions were unclear as three patients also received ureterolysis immediately after diagnosis.^9^ One patient was also treated with a percutaneous nephrostomy at some point during their treatment.^9^ Renal function improved by over 50% compared with baseline in 12 (66.7%) patients; whereas, two (11.1%) displayed minimal changes to their creatinine levels, and three (16.7%) required at least one episode of dialysis.^9^ Two (11.1%) also suffered relapse after ureteric stent insertion and a combination of medical treatments.^9^ Of note is that the authors did not reveal exactly what treatment strategies were used in those whose renal function improved, stayed the same or necessitated dialysis. As a result, delineating which options led to better outcomes is challenging.^9^ In addition, the exact reasons as to why different treatments were used was again not discussed, and the sample of patients studied was very small.^9^ This study therefore suggests that although treatment methods can be effective, determining which methods are most effective requires more strict treatment allocation and larger cohorts of patients.

A study consisting of five patients demonstrated that laparoscopic ureterolysis without omentoplasty can yield excellent results for patients. None of the patients complained of symptoms, required corticosteroids or underwent haemodialysis during a mean follow‐up of 31 months.^10^ Each of these patients underwent unilateral surgery, and four (80%) had been treated with corticosteroids prior to surgery.^10^ Two (40%) additionally received treatment with tamoxifen.^10^ This study therefore suggests that medical treatment prior to surgery can successfully treat OU secondary to RPF. The study is highly susceptible to selection bias however, given its small cohort size. Furthermore, because it does not compare outcomes with other treatment methods, laparoscopic ureterolysis without omentoplasty could not be said to be superior to other strategies.

### 3.4 Studies reporting positive outcomes across a variety of treatment strategies

Liao et al. performed a relatively large retrospective study in a population of Chinese RPF patients. One hundred and ten (77.5%) patients received corticosteroids either alone or in combination with immunosuppressants, and two (1.4%) were treated with hydroxychloroquine alone.^11^ Sixty‐nine (48.6%) patients underwent ureteric stenting, nine (6.3%) underwent ureterolysis and a further nine received either a nephrostomy or underwent nephrectomy.^11^ Sixty‐three (44.4%) patients received medical therapies in combination with a form of surgery, and nine (6.3%) received no treatment at all.^11^ Excellent outcomes were reported across the whole cohort, with all patients showing improved symptoms and decreased ESR and creatinine levels during follow‐up.^11^ In addition, 89% of patients displayed a greater than 25% reduction in their peri‐aortic mass.^11^ It should be noted that the follow‐up times and range of disease duration varied significantly among the cohort (Table 1), reducing the validity of the study's conclusions regarding long‐term outcomes.

The radiological and biochemical outcomes following treatment were well documented in this study. Because outcomes among patients receiving different treatments were not compared however, we cannot conclude which methods worked best among this cohort.^11^ In addition, little information was provided regarding why different medical and surgical treatments were selected, preventing readers from considering the reasoning behind treatment choices.^11^ Because all patients showed symptomatic and biochemical improvements, each of the strategies may be appropriate in individual circumstances and remain as viable options should patients suffer disease progression or relapse. Because positive outcomes were also reported among patients who had no treatment, delaying the start of treatment may be best in some with milder disease and/or who are at high risk of significant treatment side effects or complications.

Yachoui et al. performed a study in which 26 RPF patients received either medical treatment alone (26.9%), surgical treatment alone (26.9%) or a combination of medical and surgical options (42.3%) between 1998 and 2013.^12^ One (3.8%) patient received no treatment.^12^ Of those treated medically, 10 (38.4%) received steroids alone, two (7.7%) received steroids with tamoxifen, three received steroids with a DMARD (11.5%) and three (11.5%) received tamoxifen alone.^12^ Fourteen (53.8%) patients underwent ureteric stenting, seven (26.9%) underwent ureterolysis, two (7.7%) underwent neocystostomy, two (7.7%) underwent nephrostomy insertion and one (3.8%) received a ureterostomy.^12^

Excellent overall outcomes were reported during the five‐year follow‐up period.^12^ Fifteen (83.3%) patients who were started on medical therapy were able to discontinue their treatment, 12 (85.7%) patients were able to have their ureteric stents removed and no episodes of disease relapse were reported.^12^ Twenty‐four (92.3%) patients had CT imaging around the time of follow‐up, which displayed full resolution in six and improvement in 10 cases.^12^ Much like Liao et al., this study suggests that each of the strategies employed may result in highly effective treatment. Because the treatment strategies were not directly compared and the exact reasons for treatment choice were not discussed however, conclusions about treatment superiority and when to employ various methods remain unclear.

Liu et al. explored the outcomes among 30 patients who initially received either medical or surgical treatments alone or a combination. Corticosteroid monotherapy was initiated in five patients following which three required ureteric stenting to preserve kidney function.^13^ Twenty‐four patients received surgical interventions alone.^13^ Ureteric stenting was performed in 17 (56.7%) cases, four (13.3%) received ileal reconstruction, two (6.7%) underwent ureterolysis and one (3.3%) had a nephrostomy inserted. One patient received no treatment and was lost to follow‐up.^13^ Formal comparisons between treatment methods were lacking once again, preventing analysis of treatment superiority.

Although 93.3% of patients displayed improved eGFR and creatinine after treatment, a statistically significant change was not seen in the cohort's mean values after treatment.^13^ Renal function improvement was significant in those who displayed bilateral hydronephrosis at baseline.^13^ Ureterolysis and ileal reconstruction were also associated with greater improvements than ureteric stenting, though each of these treatments led to elevated kidney function.^13^ Although most patients saw improvements, this study suggests that treatment with steroids alone is likely to necessitate future ureteric stenting and that ureteric stenting alone is inferior to ureterolysis and ileal reconstruction. In addition, those with bilateral hydronephrosis and poorer renal function at baseline are more likely to see improvements following treatment than those with milder disease. This study therefore suggests that these patients may benefit from treatment earlier in the disease course and with more invasive methods.

### 3.5 Treatments with high complication rates

In one retrospective observational study by Chua et al., 24 RPF patients underwent either double‐J stenting or percutaneous nephrostomy, of which 17 (70.8%) subsequently underwent ureterolysis and two (8.3%) received extra‐anatomical stents^14^; 82.4% of those who underwent ureterolysis did not require additional stents or nephrostomies by the end of the follow‐up period.^14^ Many patients, however, continued to have chronic kidney disease (CKD) Stage 3a following ureterolysis, and five required a subsequent nephrectomy.^14^ Furthermore, many required additional surgery to manage ongoing complications, and 41.7% reported having chronic pain by the end of follow‐up. Although ureterolysis effectively preserved kidney drainage, it failed to alleviate patients from chronic renal impairment and troublesome symptoms.^14^ This study also showed that treatment with steroids or methotrexate alone was ineffective and that azathioprine was poorly tolerated. This study therefore showed that all treatment modalities were associated with either residual renal impairment, ongoing symptoms and/or significant treatment side effects. This contrasts with most of the other studies, which generally report high proportions of patients with improved symptoms and renal function.

### 3.6 Studies assessing outcomes with a focus on IgG4 patients

The role of IgG4 in the aetiology of RPF is becoming increasingly recognised, and it is possible that many patients who have been diagnosed with idiopathic disease may be re‐classified as having IgG4 related RPF.^15^, ^16^ Given the success of steroids and biologic agents such as rituximab in patients with IgG4 related RPF, determining whether a patient has idiopathic or IgG4 related disease may prevent unnecessary procedures and improve overall outcomes.^16^

Forestier et al. performed a retrospective observational study of 18 patients in which 11 (61.1%) had ureteric obstruction.^15^ Eight (44.4%) and four (22.2%) patients were shown to have imaging features of possible and highly likely IgG4‐related disease, respectively.^15^ All eight patients with possible IgG4‐related disease were treated with corticosteroids alone, following which three relapsed after steroid tapering and required immunosuppressants.^15^ One of the patients with highly likely IgG4‐related disease required additional treatment with methotrexate, and two others required bilateral ureteric stenting after receiving steroids.^15^ The treatments and outcomes of the idiopathic cases were not discussed.^15^ Overall, this study suggests that many patients with possible and likely IgG4‐related disease will require additional treatment after receiving steroids alone.^15^ Combining steroid treatment with other interventions may therefore serve these patients best, but because outcomes following this and other strategies were not formally compared, the strength of this conclusion is limited.^15^

Forestier et al. also found that there were no specific imaging appearances that could reliably differentiate between idiopathic and IgG4‐related RPF and concluded that tissue biopsies remain necessary to make this distinction.^15^ Numerous methods of achieving tissue biopsy exist, including peri‐operatively as part of ureterolysis.^17^, ^18^ Because this study showed that many patients required additional treatment after receiving corticosteroids,^15^ it may be best to take biopsies as part of interventions such as ureteric stenting or ureterolysis in those whose imaging suggests IgG4‐related disease.

A study consisting of IgG4‐related RPF patients reported positive responses, and no instances of relapse among seven (70%) patients treated with corticosteroids.^19^ Resolution of hydronephrosis was seen in all five patients following steroid treatment.^19^ Out of the seven treated with steroids, one underwent bilateral‐ureteric stenting, another had a left‐sided resection of a ureteric mass and another underwent resection of an abdominal aneurysm.^19^ None of the three patients managed without steroids required surgery or showed evidence of either disease remission or relapse.^19^ Although steroid treatment was effective in many of these patients, additional treatment with medical or surgical interventions was necessary in a significant proportion of the cohort.^19^ Management with a combination of treatments and/or regular follow‐up that can detect complications early may therefore be necessary. The study's small sample size, however, prevents any significant comparisons between different treatment regimens from being made and therefore limits the strengths of its conclusions.^19^

## 4 DISCUSSION

This review has analysed literature focusing on the treatment and management of RPF patients with associated OU published between 2009 and 2022. The included studies came from a wide variety of countries and incorporated patients that, on average, represent the demographics and symptomatology seen in the RPF population. The compiled outcomes across the studies are therefore likely to be representative of the average RPF population. Our search failed to find any randomised control trials (RCTs) comparing treatment outcomes between RPF patients with OU receiving medical, surgical or a combination of treatments. All included studies were retrospective observational studies, retrospective descriptive studies or case series.

Five studies suggested that a combination of medical and surgical treatments was associated with positive outcomes.^5^, ^6^, ^7^, ^8^, ^9^ Just one of these studies directly compared the prognosis of patients receiving different treatment regimens.^5^ The other four publications involved giving steroids and/or performing ureteric stenting to all or a large majority of the patients, which mostly resulted in positive outcomes. In addition, the studies by Forestier et al. and Chiba et al. showed that large proportions of the patients who received medical treatment alone required subsequent ureteric stenting.^15^, ^19^ These studies also suggested that those treated with medications alone should be followed up regularly so that treatment failure and relapses can be detected and treated early. Bergero et al. also reported positive outcomes among five patients treated surgically, of which four also received treatment with corticosteroids.^10^ There is therefore evidence that combining medical and surgical treatments can manage RPF patients with associated OU. A lack of formal comparisons of the outcomes between specific treatment protocols assigned to large, randomised cohorts limits conclusion that this strategy is superior to other treatment methods.^10^

Liao et al., Yachoui et al. and Liu et al. showed high levels of treatment success can be achieved regardless of the specific regimens employed.^11^, ^12^, ^13^ They suggest that treatment methods should be chosen after considering patients' individual characteristics to minimise their complications and side effects. Two small studies analysed outcomes in patients with IgG4‐related RPF, with one highlighting the continued need for retroperitoneal biopsy to identify this subgroup.^15^, ^19^ Because surgical biopsies can be combined with procedures such as ureterolysis, patients who need urinary decompression may be best served by having these performed simultaneously. The surgical approach used to perform ureterolysis was only mentioned in three studies, and none reported using robotic assistance.^7^, ^9^, ^10^ Laparoscopic ureterolysis has been shown to reduce the length of hospital stays and the need for blood transfusions but was no better at resolving ureteric obstruction compared with open ureterolysis.^20^ The first reported case of robotic‐assisted ureterolysis was published in 2006, and many advantages of this approach compared with open surgery have been found.^21^, ^22^ There is no consensus on the advantages of robotic versus laparoscopic ureterolysis however, and future studies comparing these approaches may provide greater clarity on the optimum management of RPF.^22^ There is currently a paucity of literature that formally analyses treatment outcomes following treatment with specific treatment regimens among RPF patients with OU. In addition, the studies were all based in single centres, and many had small sample sizes, which limits the external validity of their results. More large analytical studies are certainly required to truly conclude which initial and subsequent treatment methods are best for RPF patients with secondary OU.

The studies also commonly did not reveal why certain treatment options were chosen and none described using any specific treatment protocols. Many of the patients who received medical treatment will have received multiple different medications and vice versa for those who had surgery. This prevents direct comparisons between specific treatment groups from taking place, particularly in studies with small sample sizes. Because the severity and symptoms of RPF are often highly variable, it is possible that a nuanced approach to treatment will always be required. Future studies with large numbers of patients assigned to specific treatment protocols will provide much greater clarity on how best to manage RPF patients with OU. Although rates of disease relapse and treatment failure were often low, correlations between outcomes, demographic and clinical factors were not assessed in the studies reviewed. More data regarding risk factors for treatment failure and disease relapse would therefore also be useful in optimising treatment choices and planning follow‐up.

## 5 CONCLUSION

Following a literature search, 12 studies were found that reported treatment outcomes among RPF patients with OU. Many studies concluded that a combination of medical and surgical treatments, most commonly steroids and ureteric stenting, resulted in positive treatment outcomes. Others noted the possibility of good outcomes from a variety of treatment strategies and highlighted the array of options available. Because no large RCTs or multi‐centre analyses were included and just one study formally compared outcomes between patients receiving different treatments, conclusions regarding which treatment strategies were superior were significantly limited. More research analysing treatment outcomes is required to gain greater clarity regarding the optimum management strategies.

## AUTHOR CONTRIBUTIONS

**Charles Carey:** main author, literature search, data collection, data analysis, manuscript write up; **Garard Gumurthy:** data collection; **Richard Napier‐Hemy:** supervisor; **Bachar Zelhof:** supervisor.

## CONFLICT OF INTEREST STATEMENT

The authors declare no conflicts of interest.

## APPENDIX A

Figure A1 shows the literature search strategy and number of results acquired from Medline, Embase, Cinahl and the Cochrane library to find the publications assessed during this review.
